# Association of decreased lymphatic vessel density with postoperative eyelid edema

**DOI:** 10.3389/fopht.2025.1689146

**Published:** 2026-01-07

**Authors:** Christine L. Bokman, Roy P. Yu, Shaili S. Davuluru, Rasika Sudharshan, Kristen E. Park, Sarah Guo, Joy Li, Preeya Mehta, Alice Shen, Jessica R. Chang, Alex K. Wong, Young-Kwon Hong, Sandy X. Zhang-Nunes

**Affiliations:** 1 Department of Ophthalmology, Keck School of Medicine, University of Southern California, Los Angeles, CA, United States; 2 Department of Surgery, Keck School of Medicine, University of Southern California, Los Angeles, CA, United States; 3 Department of Surgery, Rutgers New Jersey Medical School of Medicine, Newark, NJ, United States; 4 Department of Ophthalmology, Kaiser Permanente Los Angeles Medical Center, Southern California Permanente Medical Group, Los Angeles, CA, United States

**Keywords:** edema, eyelid, blepharoplasty, post-operative, lymphatic, density

## Abstract

**Purpose:**

We sought to investigate whether variations in lymphatic morphology are associated with postoperative eyelid edema in patients who underwent upper eyelid blepharoplasty.

**Methods:**

This was a prospective study in which nine upper eyelid skin samples were stained with Podoplanin to immunochemically mark lymphatic vessels. Lymphatic vessel area, density, and perimeter were calculated from averaging 38 slices across 3 cuts of eyelid skin. Corresponding postoperative patient photographs were graded by four physicians with a standardized postoperative edema grading scale that ranged from zero (no edema) to three (severe edema). Patients were classified as having clinically significant eyelid edema (CSEE) if they received an edema grade greater than zero 90 days postoperatively or a grade of three at any time point.

**Results:**

Anti-podoplanin staining demonstrated that there was significantly lower lymphatic vessel density among patients with severe edema (8.00 ± 1.67 vessels/mm^2^) compared to patients with mild or no edema (12.14 ± 1.93 vessels/mm^2^, *p* < 0.05). Lymphatic vessel area and perimeter did not reveal any significant associations with postoperative edema grades.

**Conclusions:**

In patients undergoing an upper eyelid blepharoplasty, severe postoperative swelling was significantly associated with lower lymphatic vessel density, but not with lymphatic vessel area or perimeter. These findings suggest that the lymphatic network of the eyelid could play a role in the degree of postoperative swelling.

## 1 Introduction

While postoperative edema after eyelid surgery is to be expected, clinical observation has demonstrated unpredictability in the degree and duration of edema. Persistent eyelid edema is a feared complication of eyelid surgery. Prolonged edema leads to fibrosis and lasting disruptions in local fluid homeostasis if not alleviated in a timely manner. Fluid may spread from the eyelid to conjunctiva, causing persistent chemosis and visual deficits. Although most cases of postoperative eyelid edema resolve with time, there are instances in which edema does not clear spontaneously and requires medical or surgical treatment (1). Unfortunately, there are few evidence-based guidelines on mitigating or preventing postoperative edema (2).

The etiology may be due to a multitude of underlying causes and concurrent complications (1, 3). In terms of histological factors, the rich network of lymphatics within the eyelid skin has been shown to play a role in eyelid edema, suggesting that the disruption of lymphatics during surgery is one contributing factor (4–9). Several studies have established the intricate anatomical network of lymphatic vessels in eyelid skin (10–13). This network can be delineated into vascular and lymphatic systems by identifying endothelial proteins specific to the individual capillaries. Podoplanin (PDPN), for instance, is a unique transmembrane protein that can serve as a marker to the lymphatic system in immunohistological analyses (14, 15). Several studies confirm the high specificity and sensitivity of podoplanin alone to identify and outline lymphatic tissue, without non-specific staining of myoepithelial cells (14). Five studies to date have investigated the histopathological changes in eyelid skin from patients with dermatochalasis, including variations in lymphatic morphology (16–20). We found only one study detailing lower eyelid anatomy and its correlation to postoperative edema, which found that concurrent injury to both the superficial and deep lymphatic drainage systems led to postoperative edema (21). However, there exists a knowledge gap regarding the relationship between lymphatic morphology of the upper eyelid and degree of postoperative edema, including persistent or severe edema.

As such, the purpose of our study was to investigate and quantify lymphatic associations with postoperative eyelid edema in patients who underwent upper eyelid surgery. The present study directly compares lymphatic findings of eyelid skin with clinical variations in eyelid edema experienced by patients in the postoperative course.

## 2 Methods

This was a prospective histopathological study. Specimens were collected from nine patients who underwent upper eyelid blepharoplasty by two surgeons (S.Z.N. and J.C.) during a one-year period from January 2017 through January 2018. Suture type for lid closure was 6–0 plain gut across all cases, with each surgeon employing identical technique for skin tissue excision. Exclusion criteria included any underlying conditions that may affect eyelid swelling (including thyroid eye disease, hereditary angioedema, floppy eyelid syndrome, and blepharochalasis), use of systemic diuretic medications, any recorded allergies to postoperative medications, and lack of postoperative photographs.

The study adhered to the tenants of the Declaration of Helsinki. Institutional review board (IRB) approval was obtained from the University of Southern California IRB. The study was HIPAA-compliant with the protection of individually identifiable health information. All patients were informed of the risks and benefits associated with the surgery and provided a written consent for surgery. Photo consent for figures was also obtained and is on file at the University of Southern California.

**Figure 1:**
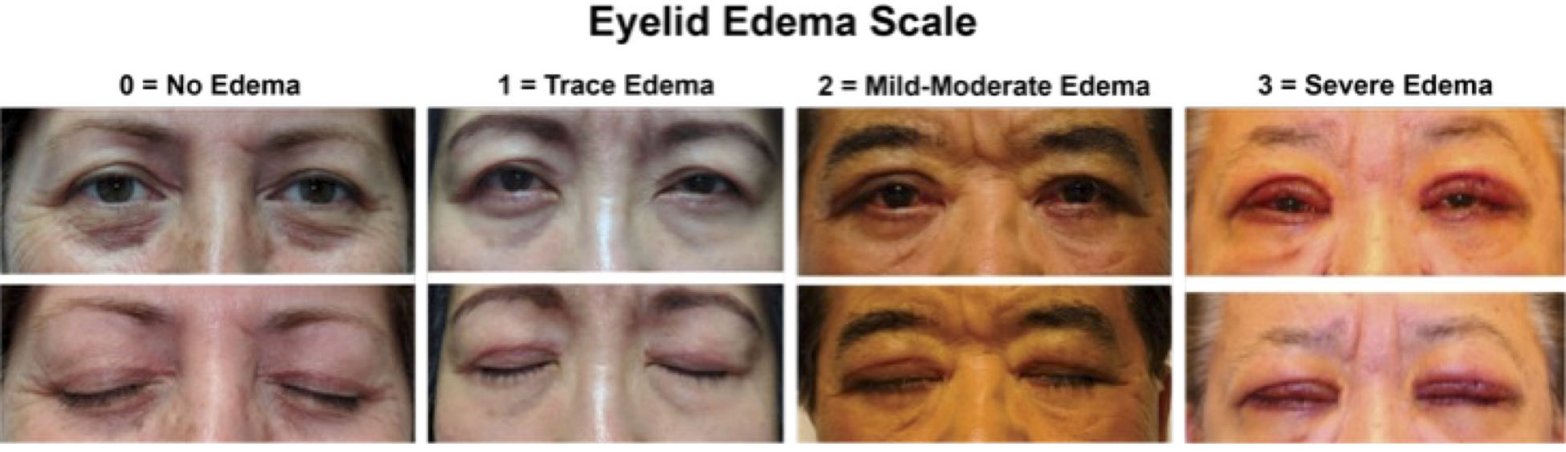
Zhang-Nunes photographic postoperative eyelid edema grading scale, ranging from 0 to 3, with 0 = no edema, 1 = mild edema, 2 = moderate edema, and 3 = severe edema.

In order to assess postoperative edema, a photographic postoperative eyelid edema grading scale previously described in Zhang-Nunes et al., was utilized. Briefly, it is defined as follows: 3 indicating severe edema or edema that extended to either the eyelid margin or brow; 2 indicating mild to moderate edema or edema that extended beyond the incision line but not all the way to the eyelid margin or brow; 1 indicating trace edema or edema confined to the incision line; and 0 indicating no edema (**Figure 1**) (21).

Using this reference scale, four expert physicians – two oculoplastic surgeons, one oculoplastics fellow, and one ophthalmology resident – graded postoperative photographs. All physicians graded every photograph of each patient included in the study. Patient photographs were deidentified, assigned a random number, and contained in an online database. Graders were masked to patient information, including patient demographics, surgeon, and follow-up period.

At least three postoperative photographs were graded per patient, as patients were photographed at postoperative week one, postoperative month one, and postoperative month four. If a patient received a postoperative eyelid edema grade of 3 at any time point postoperatively or any grade that was non-zero more than 90 days postoperatively, they were classified as having clinically significant eyelid edema (CSEE).

Standard demographic information was also collected for each patient, including age, gender, ethnicity, and body mass index (BMI). Age and BMI were analyzed as continuous variables while gender was analyzed as a binary value. Ethnicity was also analyzed as a binary value as either Asian or non-Asian. Asian ethnicity was defined as East Asian (including China, Taiwan, Japan, Korea), Southeast Asian (including Thailand, Philippines, Vietnam), South Asian (including India), and Native Hawaiian/Pacific Islander. Non-Asian ethnicities included Black, Hispanic, White, and Other/Unknown.

Eyelid specimens were immunostained with Podoplanin (sc376695, Santa Cruz Biotechnology, Inc., Dallas, TX) to immunohistochemically mark lymphatic vessels. Specimens were deparaffinized, underwent antigen retrieval with sodium citrate buffer, incubated overnight with the primary antibody (1:100) at 4 °C, washed, incubated with a suitable secondary antibody, stained with immPACT DAB Substrate (Vector Laboratories, Inc, Burlingame, CA), and counterstained with hematoxylin diluted 1:4.

Podoplanin stained vessels in the eyelid dermis were visualized on ImageJ (U.S. National Institutes of Health, Bethesda, Maryland). Three high-power fields were randomly selected per specimen. For all vessels within those three high power fields, lymphatics morphology was quantitated by measuring lymphatic vessel area, density, and perimeter. Lymphatic vessel area was calculated by measuring the total area within the podoplanin stained vessels per high-power field image. Lymphatic vessel density was calculated from the absolute lymphatic number normalized to the dermal area measured (number of lymphatics/mm^2^). The lymphatic perimeter was quantified by totaling the measured diameter, or the length of the vessel border, of the podoplanin stained vessels per high-power field image.

All measurements were expressed as mean +/- standard deviation. Variables were analyzed using one-way ANOVA and student’s t-test. Statistical significance was defined as *p* < 0.05. Analyses were performed with SPSS version 28.0.1 (SPSS, Inc, Chicago, IL).

## 3 Results

Skin specimens obtained from nine patients undergoing bilateral upper eyelid blepharoplasty were analyzed. The subject age range was 25–84 years (mean age = 64 years). Additional demographic data are listed in **Table 1**. Of the 9 specimens, 1 had no edema (edema score = 0), 3 had mild edema (edema score = 1), 3 had moderate edema (edema score = 2), and 2 had severe edema (edema score = 3). Cohen’s kappa statistic demonstrated a near perfect agreement between graders in our current and prior studies (κ=0.80 and κ=0.81, respectively), establishing high interrater reliability with this grading scale (20).

**Table 1:** Demographic data of cohort.

Demographics	n (%) or mean ± SD
Age (years)	64 ± 16.2
Female sex	65
Asian Ethnicity	44
BMI (kg/m^2^)	29.1 ± 6.3

**Figure 2:**
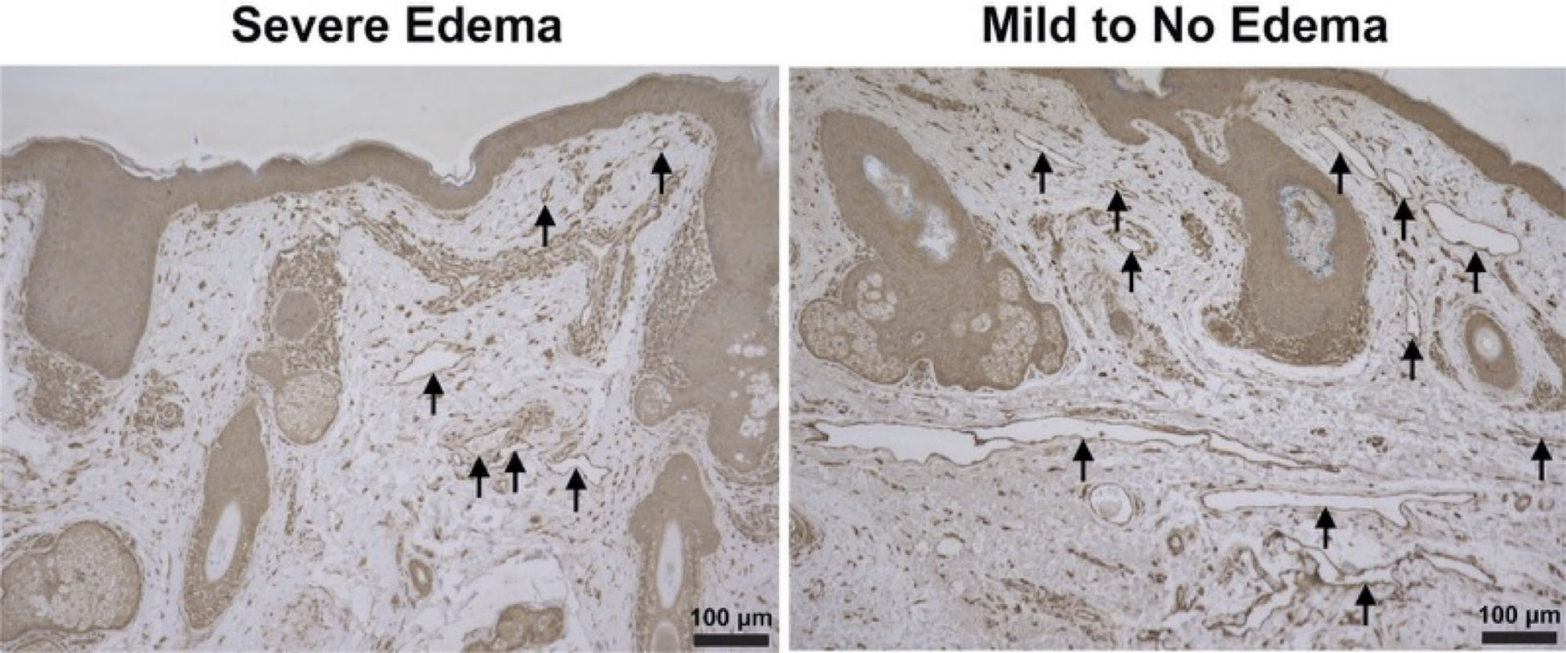
Podoplanin immunohistochemistry staining of eyelid lymphatic vessels. Left: Skin sample from a patient who had postoperative edema, grade of 3. Right: Skin samples from a patient with no postoperative edema, grade of 0. Stain: Podoplanin (sc376695, Santa Cruz Biotechnology, Inc., Dallas, TX). Magnification 10x.

**Figure 3:**
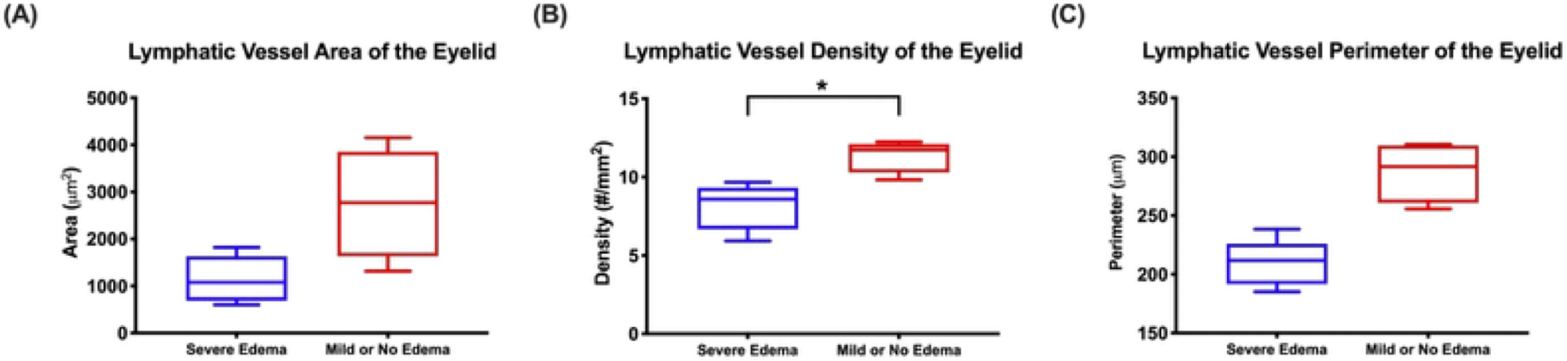
Quantification of lymphatic vessel morphology in patients with severe postoperative eyelid edema versus patients with mild or no eyelid edema versus **(A)** lymphatic vessel area, **(B)** lymphatic vessel density, and **(C)** lymphatic vessel perimeter.

**Table 2** presents information on lymphatic area, density, and perimeter. Podoplanin staining demonstrated that lymphatic vessel density was significantly lower among patients with severe edema (8.00 ± 1.67 vessels/mm^2^) compared to patients with mild or no edema (12.14 ± 1.93 vessels/mm^2^, *p* = 0.011) (**Figure 2**). The average lymphatic area among patients with severe edema was also less than that of patients with mild or no edema, but this did not reach statistical significance (1145 ± 496.9 μm^2^ vs. 2753 ± 496.9 μm^2^, *p* = 0.064).There was also no statistically significant difference between patients with severe edema compared to patients with mild or no edema in terms of lymphatic perimeter measurements (209.3 ± 19.87 μm vs. 287.3 ± 26.24 μm, *p* = 0.71) (**Figure 3**).

**Table 2:** Summary of lymphatic morphology values.

Lymphatic Vessel Metrics	Severe edema (n = 2)	Moderate edema (n = 3)	p-value* (severe vs. moderate edema)	None or mild edema (n = 4)	P-value*
Area (μm^2^)	1145 ± 496.9	1066 ± 660.3	0.625	2753 ± 496.9	0.064
Density (vessels/mm^2^)	8.00 ± 1.67	8.19 ± 2.0	0.893	12.14 ± 1.9	0.011
Perimeter (μm)	209.3 ± 19.9	216.0 ± 20.5	0.685	287.3 ± 26.2	0.710

*P-values are in reference to the severe edema group as the comparison group.

## 4 Discussion

The eyelid contains a delicate network of lymphatics and blood vessels. Several studies have demonstrated that in the upper eyelid, there are distinct lymphatic plexuses in the preorbicularis space and pre- and post-tarsal anatomical layers, which run parallel to the fibers of the orbicularis muscle with few communicating channels in between (10–13). Therefore, it is not uncommon that surgery results in varying degrees of postoperative edema. Despite this observation, there is limited evidence exploring histological characteristics of eyelid skin, including lymphatic morphology, and their correlation with postoperative edema.

We found that in patients undergoing an upper eyelid blepharoplasty, severe postoperative swelling was significantly associated with lower lymphatic vessel density when compared to patients who had mild or no edema. This finding suggests that the lymphatic network of the eyelid dermis may play a role in the degree of postoperative swelling after surgery.

Prior research has focused on lymphatic histopathology in dermatochalasis versus normal skin samples. Agliano et al. found that there was a statistically significant greater lymphatic area in patients with dermatochalasis compared to controls, but did not find any association in terms of lymphatic density (15). The patients in our study had varying degrees of dermatochalasis. While we did not control for the amount of dermatochalasis or age, Kashkouli et al. found no difference in lymphatic density between patients younger than and older than 50 years of age (20). Some studies including Nishioka et al. show a decrease in area percentage of lymphatic vessels with increase in patient age, an analogous metric to the lymphatic density defined in our study. This study further corroborates our findings of improved wound edema with increased lymphatic vessels in a given area. Further studies are needed to establish if age correlates with lymphatic density and may have potentially influenced our results (17).

The present study did not find an association between lymphatic area or perimeter with postoperative edema. Both Nagi et al. and Karnaz et al. found that lymphangiectasia, or dilation of the lymphatics, is a hallmark of lymphostasis and is more common in patients with dermatochalasis (18, 19). Our patients therefore may not have demonstrated an association between lymphatic area and postoperative edema because they all had dermatochalasis to varying degrees, which may cause dilated lymphatic vessels at baseline. Lymphatic density may be a more appropriate marker for postoperative edema as density is based on the number of lymphatics normalized to the area measured and not the size of the lymphatics, which may be influenced by a number of factors. Our findings further suggest that the network distribution of lymphatic vessels, reflected by density, may play a larger role in lymphatic drainage than the cumulative area of the vessels.

Postoperative edema after eyelid surgery is multifactorial in nature. Here, we demonstrate that lymphatic density may play a role. Further, tissue laxity and loss of elastic fibers have been shown to correlate with postoperative edema in inflammatory diseases like Melkersson Rosenthal syndrome and thyroid eye disease (5–7). Loss of a supportive cellular network necessary to support lymphatics may also represent a pathway by which lymphatics lose support, dilate, and influence surgical outcomes (18, 19, 22). In addition to histological factors, our previous research demonstrates that demographic factors such as Asian ethnicity is a risk factor, though BMI, thyroid status, medication use, and age are not (9). The anatomic and morphological differences in eyelid lymphatics we identified in this study may explain these risk factors that predispose a patient to postoperative edema. In terms of surgical technique, extensive cauterization and tissue manipulation, lateral canthal dissection, the use of dissolving vicryl sutures, and the extent of incorporation of the levator in lid crease formation may contribute to postoperative edema as well (22, 23). Our recent study found significantly higher rates of CSEE in patients who underwent mini crease enhancement, likely due to increased levator involvement by passage of more than 4 sutures through the muscle, and in patients who received buried rather than external vicryl sutures in lid crease formation (24). Future studies should consider these factors and others in the investigation of postoperative edema.

Eyelid lymphatics may influence postoperative edema not only on a morphological level but also on a physiological level in terms of absorption function. Agliano et al. also looked at the expression of lymphatic receptors for ET-1, a peptide involved in vasomotion, and found they were underexpressed in dermatochalasis specimens compared to controls (16). In postoperative edema, the functionality of lymphatics may play a role in addition to lymphatic morphology.

Our study is limited by the small sample size as well as important factors that were not controlled, such as sleeping position after surgery. The small sample size is due limits in patient consent, our rigorous inclusion criteria, and cost of staining. While many patients may have undergone blepharoplasty by the two surgeons during the study period, only a limited portion consented to the use of their skin tissue for this study. Several patients seen by these surgeons also had confounding factors such as thyroid eye disease, floppy eyelids, or adverse reaction to post-operative medications that excluded them from the study. Although we were unable to stratify the data or control for confounding factors such as sleeping position, all patients were operated on by the same two surgeons using the same technique, which helps minimize variation in postoperative inflammation related to suture type or surgical technique.

A future investigation will encompass a larger sample size that may allow for further sub-analysis stratifying patients by skin type, age, and various pre-operative eyelid characteristics. In expanding this study after obtaining funding support, we will transition to prospective enrollment with informed consent of follow up studies that could better inform us of the hypothesized structure-function-outcome relationship. In addition to analyzing the eyelid tissue specimen, this would involve conducting ICG lymphangiography in the eyelid prior to surgery, recording ICG flow patterns, and quantifying the data. Then, at defined post op time points (e.g., 2, 6, and 12 months), the ICG studies would be repeated. These data, skin biopsies, and lymphatic density could then be correlated to outcomes based on clinical photos. As for the staining in future larger studies, we can further validate specificity with co-staining with LYVE-1, PROX1 and other lymphatic stains. Analysis of the eyelid tissue specimen may also include a more advanced, three-dimensional analysis that can be extrapolated from multiple tissue slices.

Overall, our results present valuable information on an area of research that is currently not well understood and may guide approaches to clinical treatment for severe edema after eyelid surgery. These limited but insightful findings serve as preliminary insight on the relationship between lymphatic density and postoperative eyelid edema. However, the limited sample size and lack of control for confounding variables reduces the strength of the conclusions that may be drawn. Further confirmation is required through larger controlled studies that allow for more robust analysis of the lymphatic changes in the post-operative course and how that correlates to clinical presentation.

## Data Availability

The original contributions presented in the study are included in the article/supplementary material. Further inquiries can be directed to the corresponding author.
